# Supplementary material for: Phenotypic and Genomic Characterization of Oceanisphaera submarina sp. nov. Isolated from the Sea of Japan Bottom Sediments
Source: Life (Basel). 2025 Feb 27;15(3):378. doi: 10.3390/life15030378 (PMC11943896; doi:10.3390/life15030378)
Supplement: Supplementary file 1 [file life-15-00378-s001.zip › Supplementary_Figures.pdf]

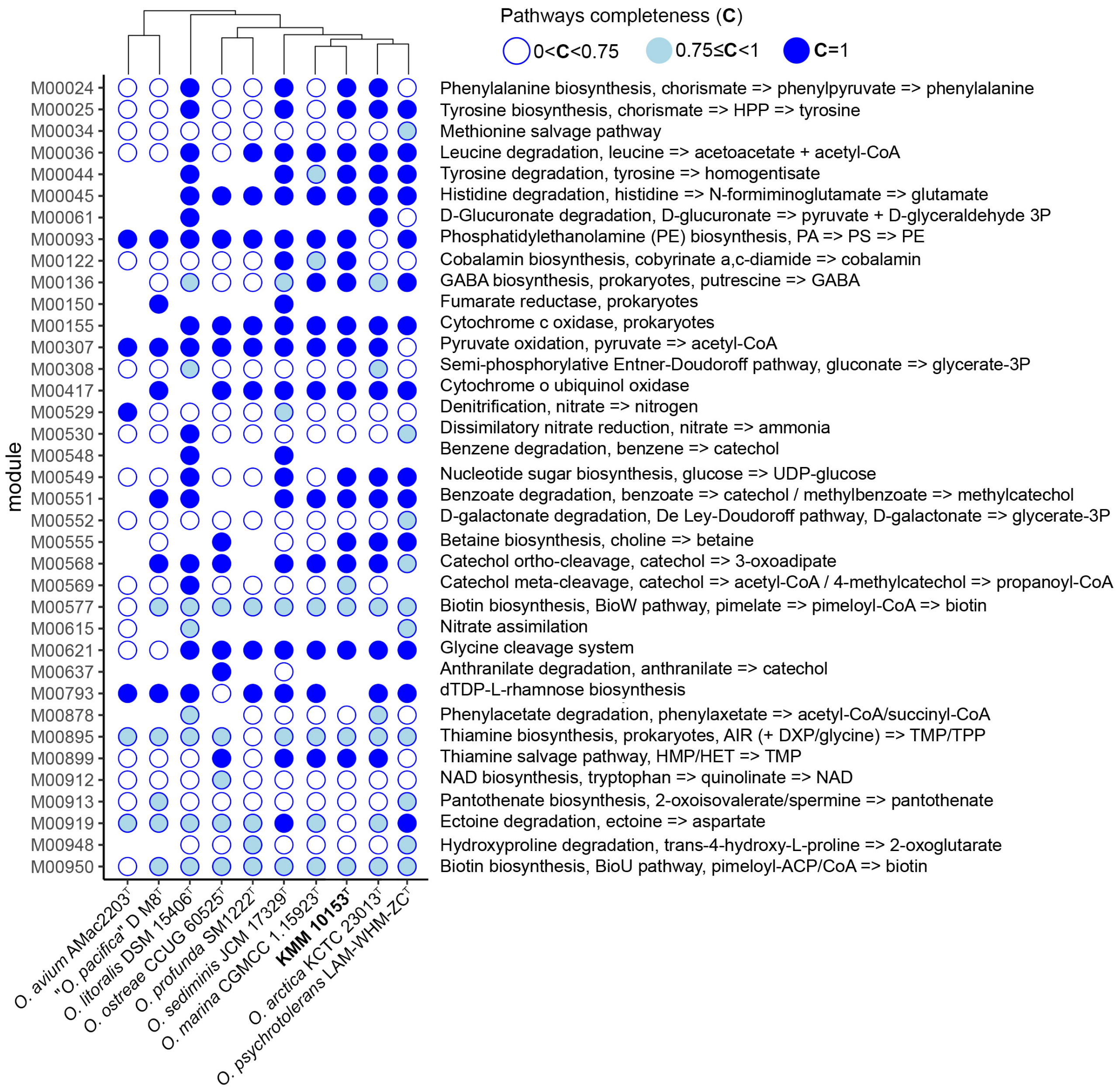

Figure S1. Discrimination of the KMM 10153^{T} and *Oceanisphaera* type strains based on completeness of predicted KEGG pathway modules

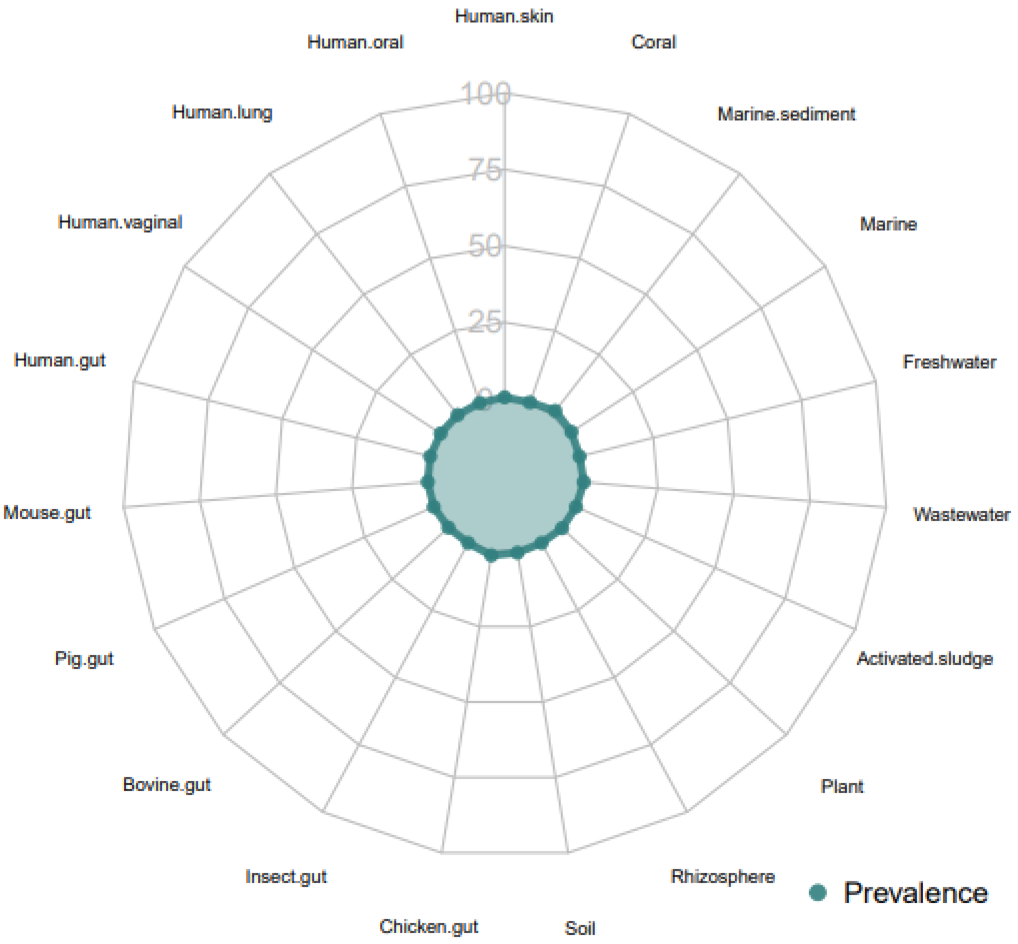

Figure S2. Habitat distribution and preference scores comparing 16S rRNA gene sequence of KMM 10153^{T} with a database of 16S rRNA gene amplicons obtained from 19 different environments by the Protologger.

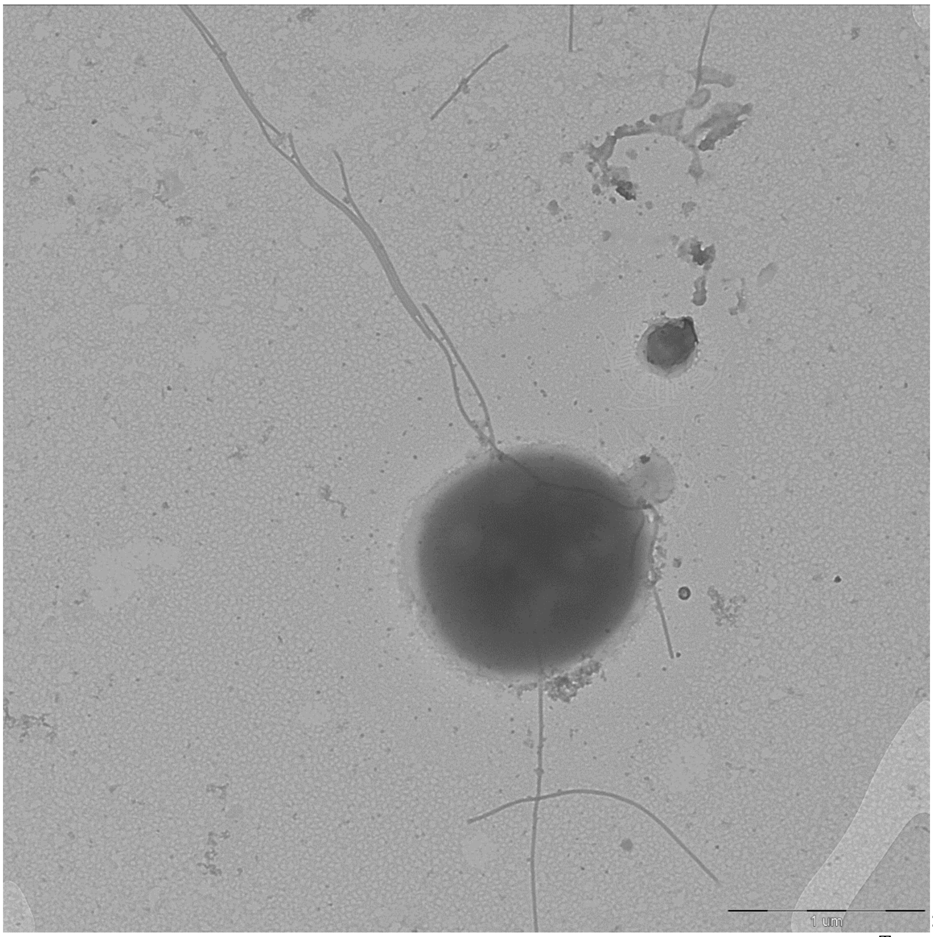

#

Figure S3. A transmission electron micrograph of strain KMM 10153^{T}. Bar, 1 μm.

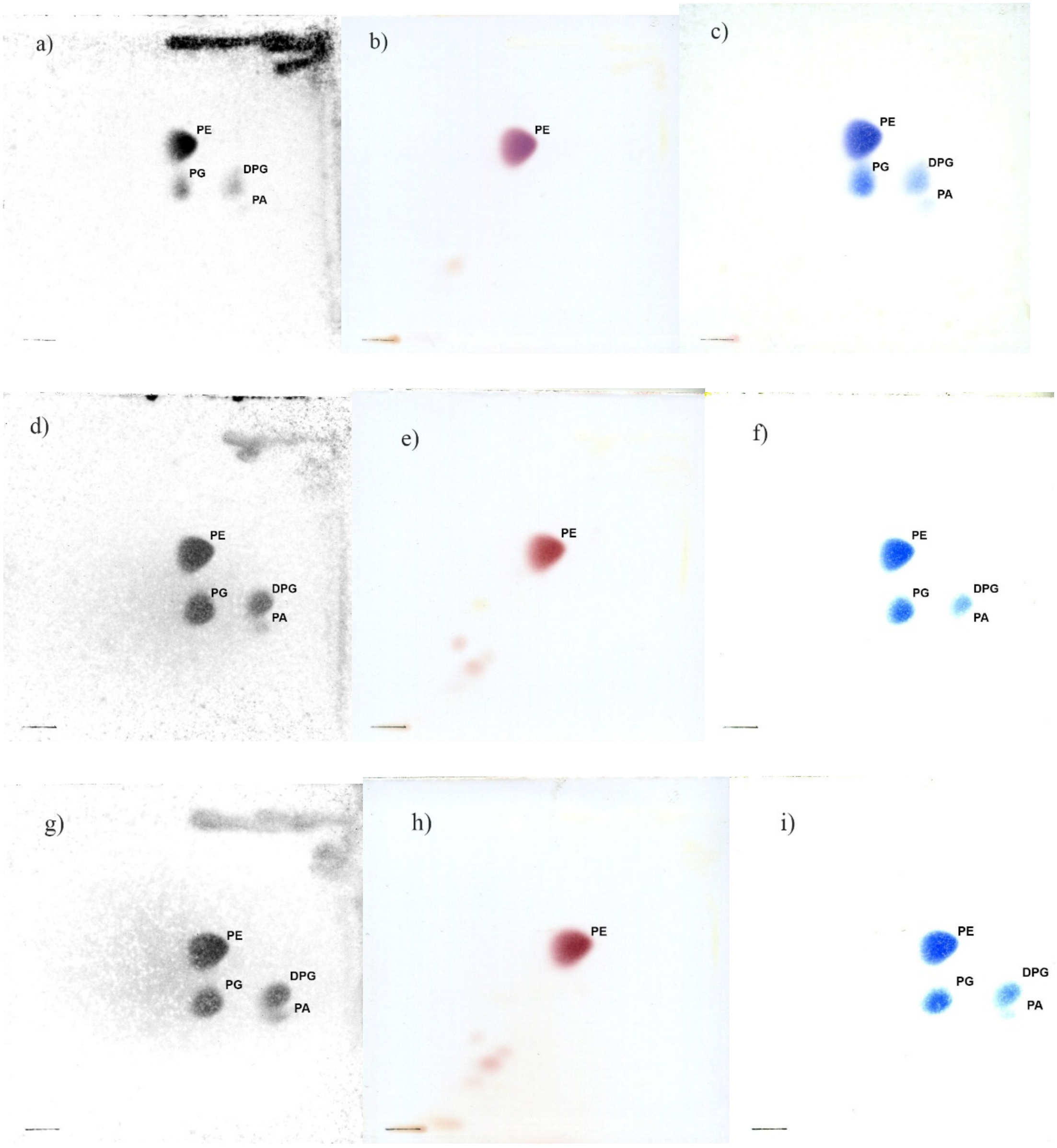

Figure S4. Two-dimensional thin-layer chromatograms of polar lipids of strains: (a, b, c) KMM 10153^T; (d, e, f) *Oceanisphaera litoralis* KMM 3654^T; (g, h, i) *Oceanisphaera arctica* KCTC 23013^T. (a, d, g), non-specific detection of lipids prepared with 10% H_2SO_4 in methanol; (b, e, h), stained with ninhydrin; (c, f, i), stained with a molybdate reagent. Abbreviations: PE, phosphatidylethanolamine; PG, phosphatidylglycerol; DPG, diphosphatidylglycerol, and PA, phosphatidic acid.
